# Supplementary material for: Gigabase-scale deletion scanning of the human genome
Source: bioRxiv. 2026 Jun 2:2026.05.29.728882. Preprint. [Version 1] doi: 10.64898/2026.05.29.728882 (PMC13251986; doi:10.64898/2026.05.29.728882)
Supplement: Supplement 4 [file media-4.pdf]

Supplementary Note 1. A constrained deletion profile with no overlapping annotated essential gene.

One beacon integration site (chr3:49419658) exhibited a significantly constrained (FDR = 0.02) post-selection deletion-length profile in haploid cells, despite the absence of an annotated essential protein-coding gene in the downstream region spanned by deletions originating at this beacon. The nested deletion series directly overlapped *TCTA, AMT, NICN1, DAG1*, and *BSN* (**Fig. SN1**; on the next page). Each of these genes is transcribed in HAP1 cells, but none are classified as essential either in the HAP1-specific CRISPR screen or in the Dependency Map dataset. Nevertheless, there are several essential genes in proximity.

Several explanations, not all of which are mutually exclusive, could account for the observed constraint: (i) One or more genes directly overlapped by the deletions is essential in HAP1 cells but was not detected as such by the CRISPR screens. (ii) The transcription start site of *RHOA* lies 5.7 kb upstream of the beacon and is oriented away from the beacon (**Fig. SN1**). RHOA fitness in HAP1 is 0.34; we consider anything below 0.5 essential. Deletions from this beacon could remove a *RHOA* regulatory element or impact *RHOA* expression through the removal of nearby expressed genes through supercoiling-mediated feedback. (iii) There is a cluster of highly essential genes further downstream that includes *MST1* (fitness 0.42) and *GMPPB* (fitness 0.12) (**Fig. SN1**). Deletions that do not directly overlap these genes could nevertheless alter their expression by removing distal regulatory elements or disrupting local chromatin architecture. Notably, there is an accessible CTCF site (black arrow in **Fig. SN1**) that coincides with the approximate boundary beyond which post-selection deletions become depleted. Disruption of that site might affect the expression of the essential genes nearby. (iv) Finally, because we test many beacon-level deletion profiles and apply an FDR of 10%, the observed constraint in this region may represent a statistical false positive.

Although this locus lacks an immediately apparent essential gene directly explaining its constrained deletion profile, it illustrates how Shred-seq may identify candidate fitness-relevant intervals that are not readily interpretable from existing gene essentiality annotations alone. Targeted follow-up experiments would be required to determine whether the observation corresponds to a missed gene-level dependency, disruption of local regulatory architecture, or a statistical false positive.

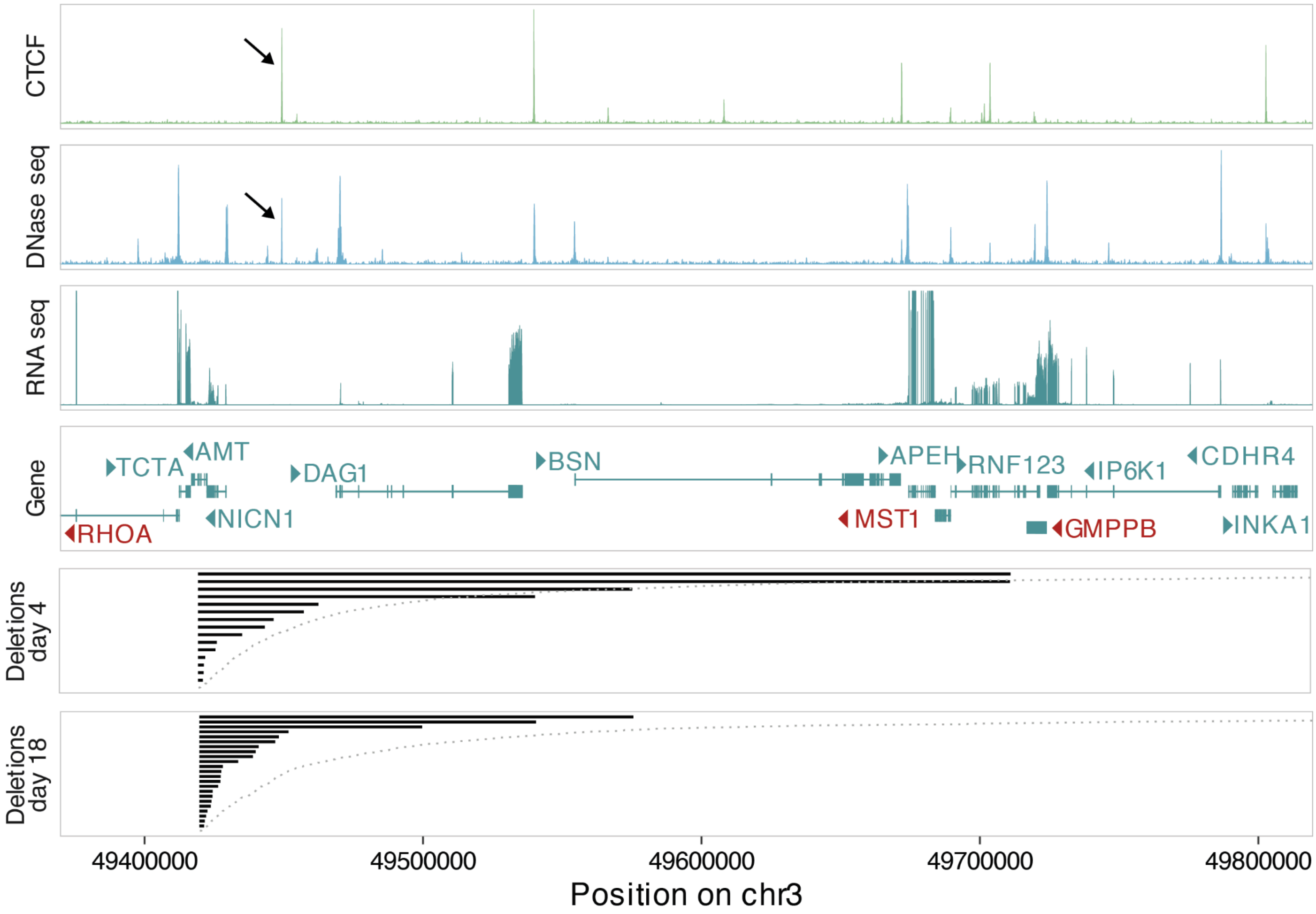

Figure SN1. Example of a beacon integration site with a constrained deletion profile, but no established essential gene downstream. Genomic features and deletions (panels) at a region on chromosome 3 bearing an integrated beacon. From top to bottom: (i) CTCF-Chip-Seq coverage track in HAP1 cells. (ii) DNase-seq coverage track in HAP1 cells. (iii) RNA-seq coverage in HAP1 cells. (iv) Exon structure and names of genes in region. Essential genes are marked in Red. Arrows indicate direction of transcription. (v) Deletions observed on day 4, with each line corresponding to length of one deletion. (vi) Deletions observed on day 18. Arrows indicate the location of an accessible CTCF site.
